# Injury of Corticospinal tract and Corticoreticular pathway caused by high-voltage electrical shock: a case report

**DOI:** 10.1186/s12883-020-01707-2

**Published:** 2020-04-13

**Authors:** Mathieu Boudier-Revéret, Ming-Yen Hsiao, Shaw-Gang Shyu, Min Cheol Chang

**Affiliations:** 1 Department of Physical Medicine and Rehabilitation, Centre hospitalier de l’Université de Montréal, Montreal, Canada; 2 Department of Physical Medicine and Rehabilitation, National Taiwan University Hospital, College of Medicine, National Taiwan University, Taipei, Taiwan; 3 Department of Rehabilitation Medicine, College of Medicine, Yeungnam University, Daegu, Republic of Korea; 4 Department of Physical Medicine and Rehabilitation, College of Medicine, Yeungnam University, 317-1, Daemyungdong, Namku, Taegu, 705-717 Republic of Korea

**Keywords:** Electrical shock, Neural injury, Diffusion tensor tractography, Corticospinal tract, Corticoreticular pathway, Weakness

## Abstract

**Background:**

We imaged the corticospinal tract (CST) and corticoreticular pathway (CRP) using diffusion tensor tractography (DTT) to evaluate the cause of muscle weakness in a patient who was exposed to high-voltage electricity.

**Case presentation:**

A 39-year-old man presented with quadriparesis after high-voltage electrical shock from power lines while working about 5.8 years ago. The electrical current entered through the left hand and exited through the occipital area of the head. The degree of weakness on bilateral upper and lower extremities was 3–4 on the Medical Research Council strength scale. Diffusion tensor imaging (DTI) was performed 5.8 years after onset. The CST and CRP were depicted by placing two regions of interest for each neural tract on the two-dimensional fractional anisotropy color map. DTT of the DTI scan showed that the bilateral CST and CRP were thinned compared to those of the healthy control subject. On the nerve conduction test, abnormal findings suggesting peripheral nerve lesion were not observed. Therefore, injury of bilateral CST and CRP seems to have contributed to our patient’s weakness after the electrical shock.

**Conclusion:**

Depiction of neural tracts in the brain using DTT can assist in the accurate and detailed evaluation of the cause of neural deficit after electrical injury.

## Background

Accidental electrical injuries can cause a variety of symptoms ranging from skin burns, damage to internal organs, to cardiac arrhythmias, respiratory arrest, and neurological injuries. While exposures to low-voltage electricity commonly occur in the household and usually do not cause significant injury to humans, exposure to high-voltage electrical shock commonly occurs in utility and construction workers and result in serious damage [1–3]. The central and peripheral nervous systems are susceptible to electrical injury because they are the route of least resistance in the human body [4]. Therefore, patients with high-voltage electrical injury frequently have neurological symptoms, such as weakness, numbness, cognitive deficits, and neuropathic pain [5]. For the evaluation of brain injury by high-voltage electricity, brain magnetic resonance imaging (MRI) is used [5, 6]. In the MRI, the lesion has a spotty appearance, confined to white matter and outside of the anatomical vascularization areas [5, 6]. However, MRI is limited in that it cannot visualize neural tract injury. Therefore, little is known about the involvement of neural tracts following electrical brain injury.

In many cases of brain injury, even patients without any abnormal findings on conventional MRI or computed tomography (CT) can have neurological symptoms induced by microscopic injury of neural tracts [7–9]. Recently-developed DTT, a derived form diffusion tensor imaging (DTI), can assess neural tract injuries at the microscopic level [7–11]. Many studies have demonstrated that DTT is a powerful evaluation tool in the assessment of neural tract injuries in patients with traumatic brain injuries, hypoxic brain injuries, degenerative brain disorders, or heat stroke [7–11].

The corticospinal tract (CST) and the corticoreticular pathway (CRP) are regarded as the most important neural tracts for voluntary movement in humans [7, 9, 11, 12]. Microscopic injuries of these neural tracts is a main cause of muscle weakness in patients with brain injuries [7, 9, 11, 12]. In the current study, using DTT, we present injuries to the CST and the CRP in a patient with muscle weakness without having any abnormal findings on MRI after exposure to high-voltage electricity.

## Case presentation

A 39-year-old man visited the Department of Physical Medicine & Rehabilitation of Yeungnam University Hospital (Daegu, South Korea) to evaluate the cause of his muscle weakness. About 5.8 years ago, the patient experienced a high-voltage electrical shock from power lines while working. He did not have any history of neurological, psychological, metabolic, or renal disorders prior to the accident. The electrical current entered through the left hand and exited through the occipital area of the head. Due to severe electrical injury of his upper limb, he received an amputation of his left upper limb above the elbow. Additionally, he received a skin transplant due to the electrical burn on the occipital area of the scalp (23 × 30 cm). Immediately after the accident, the patient presented with weakness of the bilateral upper and lower extremities (Medical Research Council [MRC] strength scale: 2–3/5). A brain and complete spine MRIs were normal at that time. He gradually recovered but still had weakness after about 5 years. In the physical examination, muscle weakness was MRC 3 for the shoulder abductors, hip flexors, and knee extensors bilaterally, and MRC 4 for the right elbow flexor, right wrist extensor, right finger flexors, and bilateral ankle dorsiflexors. Deep tendon reflexes of the bilateral upper and lower limbs were increased (3+/4) with bilateral Babinski signs. Muscle tone was mildly increased in all four limbs. He could walk independently, but appeared mildly spastic. In addition, during walking, stability was decreased. Therefore, he had difficulty going up and down stairs and was unable to run. A significant sensory deficit was not present. Signs of cerebellar or cranial nerve injuries were not observed. Additionally, pseudobulbar symptoms, such as dysphasia and dysarthria, were not shown. The Mini-Mental State Examination score was 30 points, indicating that cognitive status was within normal limits. On the nerve conduction test, no abnormal findings suggesting peripheral nerve lesion were observed. The central motor conduction times (CMCT) for the right biceps brachii (BB), abductor pollicis brevis (APB), and bilateral tibialis anterior (TA) muscles were delayed (Rt. BB 9.4 ms, Rt. APB 10.7 ms, Rt. TA 30.6 ms, Lt. TA 30.2 ms). An electrical study of the jaw and orbicularis oculi reflexes was normal. Brain, cervical, and thoracic spine MRIs taken 5.8 years after symptom onset showed no abnormal findings.

DTI data were acquired 5.8 years after the accident using a 1.5-T Philips GyroscanInteraSystem (Hoffman-LaRoche, Mijdrecht, Netherlands) equipped with a synergy-L Sensitivity Encoding (SENSE) head coil utilizing a single-shot, spin-echo planar imaging pulse sequence. For each of the 32 non-collinear and non-coplanar diffusion sensitizing gradients, we acquired 67 contiguous slices parallel to the anterior commissure–posterior commissure line. Imaging parameters were as follows: matrix = 128 × 128, field of view = 221 mm × 221 mm, TE = 76 ms, TR = 10.726 ms, SENSE factor = 2, EPI factor = 59 and b = 1000 s/mm^2^, NEX = 1, slice thickness = 2.3 mm, and acquisition voxel size = 2.3 mm × 2.3 mm × 2.3 mm.

**Fig. 1:**
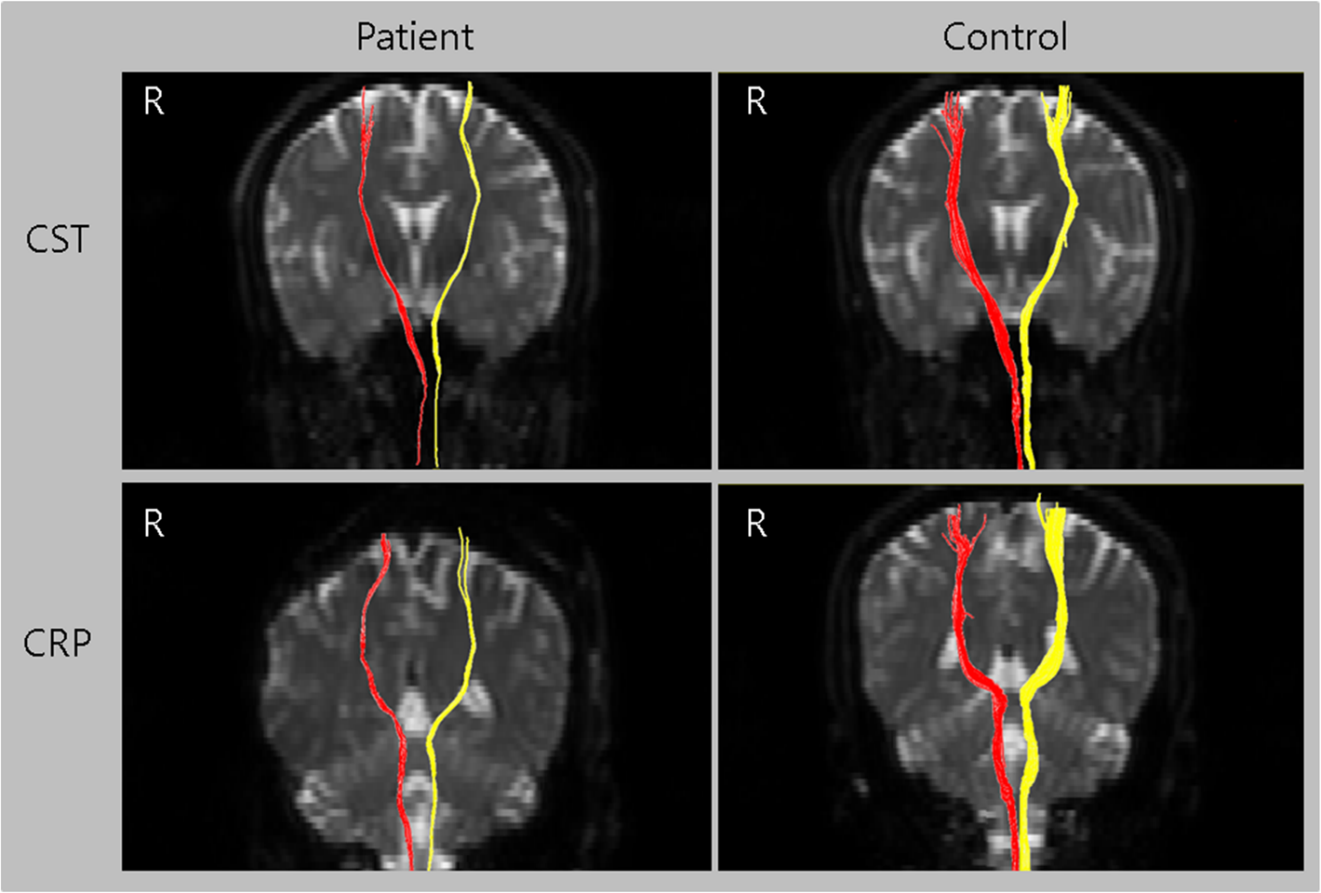
The corticospinal tract (CST) and corticoreticular pathway (CRP) of a patient with electrical injury presenting with bilateral upper and lower extremity weakness and a those of a healthy control subject (39-year-old man). A thinning of the CST and CRP in the bilateral hemispheres were observed in the patient’s diffusion tensor tractography results compared to a healthy control subject

Eddy current-induced image distortions were removed using Affine multi-scale two-dimensional registration with the Oxford Centre for Functional Magnetic Resonance Imaging of Brain (FMRIB) Software Library (FSL; www.fmrib.ox.ac.uk/fsl). DTI-Studio software CMRM (Johns Hopkins Medical Institute, Baltimore, MD, USA) was used for the evaluation of neural tracts. Fiber tracking was based on the fiber assignment continuous tracking (FACT) algorithm and multiple regions of interest (ROIs) approach. Two ROIs for each CST and CRP were placed on the two-dimensional fractional anisotropy (FA) color maps (For CST: seed ROI – the CST area of the upper pons [blue color in the pontine basis on the axial image], target ROI – the CST area of the lower pons; for CRP: seed ROI – the CRP area of the reticular formation of the medulla, target ROI – the CRP area of the midbrain tegmentum).^9^ The termination criteria were FA < 0.2 and angle >60^o^. The DTT of the patient revealed thinning of the CSTs and CRPs in bilateral hemispheres when compared to those of a healthy control subject (39-year old man) (tract volume - patient: Rt. CST = 295, Lt. CST = 285, Rt. CRP = 301, Lt. CRP = 307; control: Rt. CST = 1302, Lt. CST = 1287, Rt. CRP = 1376, Lt. CRP = 1391) (Fig. 1). The patient provided informed signed consent for participation in the study.

## Discussion and conclusions

In this study, we used DTT to evaluate the state of motor-related neural tracts (CST and CRP) in a patient with muscle weakness after exposure to high-voltage electricity and found thinned bilateral CSTs and CRPs.

The CST is known to be one of the most important neural tracts for voluntary movements and primarily controls distal muscles [12]. The CRP innervates the proximal muscles of the upper and lower limbs and the axial muscles [12]. Our patient’s bilateral CSTs and CRPs were injured, which seems to have contributed to the muscle weakness of bilateral upper and lower limbs. In addition, the delayed CMCT indicates an injury of the central nervous system (brain or cervical spine), which supports our DTT results.

The mechanisms of brain injury due to electricity are not completely defined. However, the most obvious mechanism is thermal injury [5, 6]. The large quantities of heat produced by the current passing though the body of the victim result in external and internal burns. Another proposed mechanism of brain injury is electroporation of the neurons: membrane proteins permanently change conformation and no longer maintain transmembrane ion gradients, resulting in cell death [5, 6]. The neurological complications of lightning injuries can be divided into 3 categories: immediate and transient; immediate and prolonged; or permanent, and delayed and progressive [13]. In patients with immediate and transient symptoms, the symptoms usually resolve within minutes or hours, and imaging on conventional MRI is mostly negative. In cases with immediate and prolonged or permanent symptoms, the symptoms tend to appear immediately after the electrical injury and persist for at least a few months. Several kinds of cerebral lesions, such as infarctions, hematomas, and edema, are often found. As for cases with delayed and progressive symptoms, the appearance of symptoms following electrical injury is substantially delayed from weeks to years later. However, it is suggested that there is no cause and effect relationship [13]. The delayed symptoms might have been induced by motor neuron or demyelinating diseases. Moreover, it has been proposed that exposure to electrical fields might produce effects similar to irradiation, which causes gradual structural change of active proteins and other macromolecules. In 2003, Kim et al. [14] reported that abnormalities were detected on MRI taken 14 days after an electrical injury. These abnormalities appeared as hypointensities on T1-wighted MR images and hyperintensities on T2-wighted MRI images in white matter, forming finger-like projections.

The usefulness of DTT for the evaluation of neural tract injury has been reported in several brain disorders. Additionally, in patients who did not have any abnormal findings in CT and conventional MRI after a traumatic brain injury, hypoxic brain injury, degenerative brain disorder, or heat stroke, DTT demonstrated neural tract injuries in the brain [7–11]. Regarding electrical brain injury, only one DTT study was reported. In 2017, Cho et al. reported an injury of the medial lemniscus tract in a patient with impaired proprioceptive function after a high-voltage electrical injury [15]. To the best of our knowledge, this is the first DTT study to show an injury of CST and CRP in a patient who had a muscle weakness after electrical injury.

In conclusion, our results suggest the usefulness of DTT in the assessment of neural tract injuries corresponding to the patient’s neurological symptoms even when brain CT scan or conventional MRI revealed no abnormalities. Our study is limited in that this is a single case report. Also, we did not evaluate other motor-related neural tracts, such as the rubrospinal and vestibulospinal tracts. Therefore, further studies which compensate for these limitations are warranted.

## Data Availability

All data generated or analyzed during this study are included in this published article.

## Abbreviations

DTT: diffusion tensor tractography
DTI: diffusion tensor imaging
CST: corticospinal tract
CRP: corticoreticular pathway
CMCT: central motor conduction times
BB: biceps brachii
APB: abductor pollicis brevis
TA: tibialis anterior
ROI: region of interest
FA: fractional anisotropy
